# Perfiles de expresión de los genes *ERG11*, *MDR1 y AFR1* en *Cryptococcus neoformans* var*. grubii* aislados de pacientes con HIV

**DOI:** 10.7705/biomedica.6519

**Published:** 2022-12-01

**Authors:** Isaura Torres, Juan E. Gallo, Óscar Mauricio Gómez, Álvaro Rúa-Giraldo, Juan G. McEwen, Ana María García

**Affiliations:** 1 Grupo Biociencias, Facultad de Ciencias de la Salud, Institución Universitaria Colegio Mayor de Antioquia, Medellín, Colombia Institución Universitaria Colegio Mayor de Antioquia Medellín Colombia; 2 GenEIA, Facultad de Ciencias de la Vida, Universidad EIA, Medellín, Colombia Universidad EIA Medellín Colombia; 3 Unidad de Biología Celular y Molecular, Corporación para Investigaciones Biológicas, Medellín, Colombia Corporación para Investiga. Biológicas Corporación para Investigaciones Biológicas Medellín Colombia; 4 Escuela de Microbiología, Universidad de Antioquia, Medellín, Colombia Universidad de Antioquia Universidad de Antioquia Medellín Colombia; 5 Facultad de Medicina, Universidad de Antioquia, Medellín, Colombia Universidad de Antioquia Universidad de Antioquia Medellín Colombia; 6 Facultad de Ciencias Farmacéuticas y Alimentarias, Departamento de Farmacia, Universidad de Antioquia, Medellín, Colombia Universidad de Antioquia Universidad de Antioquia Medellín Colombia

**Keywords:** Cryptococcus neoformans, criptococosis, fluconazol, azoles, farmacorresistencia microbiana, Cryptococcus neoformans, cryptococcosis, fluconazole, azoles, drug resistance, microbial

## Abstract

**Introducción.:**

El fluconazol es el antifúngico más utilizado para la prevención y el tratamiento de infecciones causadas por el género *Cryptococcus*, agente etiológico de la criptococosis. La resistencia al fluconazol en los aislamientos de *Cryptoccocus neoformans* puede hacer fracasar el tratamiento y generar recaídas de la infección.

**Objetivo.:**

Evaluar los perfiles de expresión de los genes *AFR1*, *MDR1* y *ERG11* en aislamientos clínicos de *C. neoformans* var*. grubii,* durante la respuesta *in vitro* a la inducción con fluconazol.

**Materiales y métodos.:**

Se estudiaron 14 aislamientos de *C. neoformans* var*. grubii* provenientes de pacientes con HIV, de los cuales 6 eran sensibles al fluconaol y 8 presentaban sensibilidad disminuida. Los niveles de expresión de los genes *ERG11*, *AFR1* y *MDR1* se determinaron mediante PCR en tiempo real.

**Resultados.:**

Los aislamientos resistentes al fluconazol mostraron sobreexpresión de los genes *AFR1* y *MDR1,* mientras que la expresión de los fenotipos de resistencia evaluados se mantuvo homogénea en *ERG11*, en todos los aislamientos de *C. neoformans var. grubii*.

**Conclusiones.:**

La sobreexpresión de los genes *AFR1* y *MDR1* que codifican las bombas de eflujo, contribuye a la resistencia al fluconazol en los aislamientos estudiados. Sin embargo, los patrones de resistencia que se registran en este hongo, sumado a los casos de recaídas en pacientes con HIV, no pueden atribuirse únicamente a los casos de resistencia por exposición al fármaco. Otros mecanismos podrían también estar involucrados en este fenómeno, como la resistencia emergente (resistencia mediante otros genes *ERG*) y la heterorresistencia, los cuales deben ser estudiados en estos aislamientos.

## Abstract (en)

**Introduction:**

Fluconazole is the most used antifungal drug for prevention and treatment of *Cryptococcus* spp. infections, the etiological agent of cryptococcosis. Resistance to fluconazole among *Cryptococcus neoformans* isolates can lead to treatment failure and generate relapses.

**Objective:**

To evaluate the expression profles of the *AFR1*, *MDR1* and *ERG11* genes in *C. neoformans* var. *grubii* clinical isolates during the *in vitro* response to fluconazole induction.

**Materials and methods:**

Fourteen *C. neoformans* var*. grubii* isolates recovered from HIV patients were studied, in which 6 showed sensitivities to fluconazole and 8 decreased sensitivity. The expression levels of *ERG11*, *AFR1* and *MDR1* genes were determined by real-time PCR from extracted mRNA.

**Results:**

*AFR1* and *MDR1* genes from *C. neoformans* var*. grubii* were overexpressed in fluconazole resistant isolates, whereas *ERG11* maintains homogeneous expression in all the evaluated resistance phenotypes of *C. neoformans* var*. grubii* isolates.

**Conclusions:**

The overexpression of *AFR1* and *MDR1* genes, which codify for efflux pumps, contributes to fluconazole resistance in the studied isolates. However, the resistance patterns in this fungus and the relapse cases in HIV patients cannot be attributed solely to the exposure to the drug. Heteroresistance and the emerging resistance (resistance through other *ERG* genes), might be other mechanisms involved in this phenomenon, which must be studied in these isolations.

La criptococosis es una enfermedad infecciosa de distribución mundial que abarca un amplio espectro de presentaciones clínicas. Afecta desde el huésped aparentemente inmunocompetente y sin una enfermedad subyacente, hasta los inmunocomprometidos por infección con el virus de la inmunodeficiencia humana [*Human Immunodeficiency Virus* (HIV)] o con sida, trasplante de órganos o tumores malignos.

Las manifestaciones clínicas de esta micosis pueden variar desde la colonización asintomática de las vías respiratorias, hasta la diseminación de la infección hacia cualquier parte del cuerpo humano ^1^. Se estima que cada año ocurren 220.000 casos de meningitis criptococócica entre personas con HIV-sida en todo el mundo, lo que resulta en 181.000 muertes, aproximadamente ^2^. En Colombia, la micosis alcanza una incidencia anual de 0,23 casos por cada 100.000 habitantes y, en los pacientes que padecen sida, se registra un incremento de 1,1 individuos por cada 1.000 ^3^; la cripcococosis es una de las principales causas de muerte (15 %) en esta población ^2^.

*Cryptococcus* spp. es el agente etiológico de la criptococosis. Es una levadura del filo Basidiomycota, de la cual se han descrito más de 80 especies que se clasifican como dos complejos de especies que son patógenas para los humanos: el complejo *C. neoformans y* el complejo *C. gattii*^4^. Esta levadura saprófita encapsulada se reproduce en su fase asexual mediante blastoconidias unigemantes o multigemantes asincrónicas que son infecciosas al inhalarse.

Se han descrito cinco serotipos nombrados como A, B, C, D y AD, clasificación que se basa en las diferencias estructurales de los polisacáridos de la cápsula. El complejo de especies de *C. neoformans* está, a su vez, subdividida en dos variedades conocidas como *C. neoformans* var. *grubii* (serotipo A) y *C. neoformans* var. *neoformans* (serotipo D y AD). Por otro lado, los serotipos B y C se agrupan en el complejo *C. gattii*^1^*.* De igual manera, el complejo *C. gattii* ha sido reorganizado en cinco especies: *C. gattii, C. bacillisporus*, *C. deuterogattiii*, *C. tetragattii* y *C. decagattii.* Además, se han reportado otros serotipos generados por híbridos, como el serotipo AB (*C. neoformans x C. gattii hybrid* o *C. neoformans x C. deuterogattii*) y el serotipo DB (*C. deneoformans x C. gattii*) ^4^^,^^5^*.* La infección con estas especies se ha relacionado con afección pulmonar y daño en el sistema nervioso central ^6^^-^^8^.

La epidemiología de *C. neoformans* está bien caracterizada, afectando huéspedes inmunocomprometidos e inmunocompetentes. Por el contrario, *C. gatiii* se ha considerado históricamente como agente patógeno de huéspedes aparentemente inmunocompetentes ^8^.

En la mayoría de los países en desarrollo, el tratamiento fungicida inicial combinando anfotericina B y 5-fluorocitosina (5-FC) se excluye de los protocolos por su alto costo, disponibilidad y dificultad de administración y seguimiento, a pesar de que es el recomendado por la Organización Mundial para la Salud (OMS) durante la fase de inducción para los casos de criptococosis ^9^. Lamentablemente, la disponibilidad de la 5-FC es limitada en los lugares en donde la carga y la mortalidad de la enfermedad son más altas ^8^.

La monoterapia con fluconazol (200-800 mg/día) es recomendada por la OMS como el tratamiento de elección solo durante la consolidación, el mantenimiento o la profilaxis secundaria ^9^. Sin embargo, en países en desarrollo, suele ser la única opción de tratamiento para todas las fases y etapas de la infección, a pesar de que tome más tiempo lograr la esterilización del líquido cefalorraquídeo ^10^.

El éxito de la implementación de esta terapia durante la etapa inicial de la micosis, al igual que el tratamiento en la fase de mantenimiento con fluconazol, podría ser riesgosa debido al surgimiento de cepas resistentes, cuya prevalencia es del 20 % en algunas regiones del mundo. Además, son muy frecuentes los aislamientos de este hongo obtenidos de pacientes con recaídas, con cultivo positivo, que muestran disminución de la sensibilidad a la flucitosina ^11^^,^^12^. Las fallas en el tratamiento con fluconazol de la enfermedad criptococócica, no se pueden atribuir únicamente al uso prolongado del fármaco, sino también a la resistencia emergente del agente etiológico. Además, debe considerarse el fenómeno de la heterorresistencia, el cual también ha sido reportado como un mecanismo alternativo con potencial para explicar el comportamiento del hongo ante este antifúngico ^13^.

El principal mecanismo por el cual los hongos adquieren resistencia contra los azoles, se basa en la sobreexpresión del gen *ERG11,* el cual codifica para la enzima lanosterol 14-a-desmetilasa (Erg11p), blanco de acción de estos antifúngicos. Las mutaciones en *ERG11* disminuyen la sensibilidad de esta enzima Erg11p para ser inhibida por los azoles ^14^. Venkateswarlu, *et al*. (1997), sugirieron la posibilidad de que estas proteínas transportadoras de membrana estuviesen involucradas como mecanismos de resistencia a los azoles en *Cryptococcus*. Estos autores reportaron que los aislamientos clínicos de *C. neoformans* con altos valores de concentración inhibitoria mínima (CIM) mostraban bajas concentraciones intracelulares de fluconazol, en comparación con las cepas con CIM bajas para el fluconazol ^15^.

Es así como en *C. neoformans* se han identificado dos bombas de eflujo en la membrana plasmática. La primera, es codificada por el gen *MDR1*, el cual genera para una proteína de transporte de membrana en eucariotas, relacionada con proteínas de resistencia a múltiples fármacos ^16^; y la segunda, codificada por el gen *AFR1*, el cual origina codifica para otro transportador de membrana plasmática (transportador ABC) diferente.

Las células del hongo que muestran expresión aumentada de *AFR1*, también tienen altos valores de CIM, en comparación con sus controles silvestres*.* Algunos autores han encontrado que los ratones inoculados con cepas de *C. neoformans* que muestran sobreexpresión de *AFR1*, responden de manera menos favorable al tratamiento con fluconazol, comparados con los controles infectados con la cepa silvestre. Además, la disrupción de este gen causa disminución de los valores de la CIM, y un incremento de la sensibilidad en los ratones tratados con fluconazol ^16^^-^^18^.

El objetivo de este trabajo fue evaluar los perfiles de expresión de los genes *AFR1*, *MDR1 y ERG11* en aislamientos clínicos de *C. neoformans* var*. grubii* de pacientes con HIV, durante la respuesta *in vitro* a la terapia de inducción con fluconazol.

## Materiales y métodos

### Aislamientos clínicos

Las muestras biológicas (líquido cefalorraquídeo o suero) se sembraron en agar Sabouraud glucosado sin cicloheximida; después de 24 a 48 horas de incubación a 30 °C, se observaron colonias caracterizadas como *Cryptococcus* spp., las cuales fueron replicadas en el mismo medio hasta obtener colonias aisladas. Los aislamientos recuperados durante un periodo de dos años se sometieron a criopreservación a -20 °C en medio de cultivo con agar y leche descremada, en la Unidad de Micología Médica y Experimental de la Corporación para Investigaciones Biológicas de Medellín, Colombia.

En total, se seleccionaron 14 aislamientos clínicos de *C. neoformans* var*. grubii* obtenidos a partir de dichas muestras clínicas, todas provenientes de pacientes con HIV (cuadro 1). Según la prueba de sensibilidad *in vitro y* de acuerdo con los puntos epidemiológicos de corte, 8 aislamientos mostraron disminución de la sensibilidad al fluconazol y 6 mostraron un fenotipo sensible ^19^^,^^20^.

**Cuadro 1:** Características de los aislamientos de *Cryptococcus neoformans var. grubii* empleados en el estudio

Identificación del aislamiento*	Fenotipo de resistencia	CIM* fluconazol (μg/ml)	Sub-CIM* (μg/ml)
64212	Resistente	>256	64
66992	Resistente	>256	64
64146	Resistente	>256	64
57332	Resistente	>256	64
53687	Resistente	>165	64
24198	Sensible	1	0,5
8843	Sensible	5	2
22602	Sensible	6	2
24405	Sensible	1,1	0,5
23849	Sensible	1,1	0,5
63127	Sensible	4	2
23433	Sensible dosis dependiente	32	16
18666	Sensible dosis dependiente	16	8
52550	Sensible dosis dependiente	61	32

* La concentración inhibitoria mínima (CIM) se determinó mediante el método de difusión con discos.

+ Todos los aislamientos fueron recuperados de pacientes con HIV

• Concentraciones sub-CIM usadas para el tratamiento de cada uno de los aislamientos estudiados. Los valores se asignaron según el valor de CIM previamente determinado.

### Pruebas de sensibilidad

Las pruebas de sensibilidad se realizaron mediante el método de difusión con discos de fluconazol de 25 μg (Beckton Dickinson, Spark, MD), siguiendo el protocolo establecido por el *Clinical and Laboratory Standards Institute* (CLSI), en la guía M44 propuesta por el *National Committee for Clinical Laboratory Standards* (NCCLS) ^21^. La lectura de los halos de inhibición fue documentada en el equipo BIOMIC™.

En el caso de *Cryptococcus* spp., aún no se han establecido los puntos de corte clínicos para determinar los fenotipos de sensibilidad al fluconazol, por lo cual se utilizan los puntos de corte epidemiológicos ^19^^,^^20^; estos corresponden al valor de la CIM que marca el límite superior de los aislamientos de tipo salvaje (*wild type*). Para *C. neoformans* var. grubii, se definió un punto de corte epidemiológico de 8 pg/ml; por consiguiente, los aislamientos con una CIM mayor de 8 pg/ml se consideraron con sensibilidad disminuida al fluconazol ^20^.

### Análisis de expresión

*Purificación del ARN*

Se recuperó el ARN total de los 14 aislamientos del estudio, después de su crecimiento en el medio de cultivo líquido YPD (*Yeast Peptone Dextrose*) mediante incubación a 30 ^o^C y 150 rpm durante 24 horas. Se inoculó 1 ml del cultivo del hongo en fase logarítmica (1 x 106 blastoconidias por ml, contadas en cámara de Neubauer) en dicho medio, con el fin de normalizar las condiciones de todos los aislamientos evaluados.

Los cultivos se incubaron a 30 °C y 150 rpm durante 48 horas en ausencia del antifúngico, para luego ser incubados durante 2 horas bajo concentraciones de fluconazol inferiores a la CIM ^16^ (cuadro 1). A continuación, se recolectaron las blastoconidias y se procedió a extraer el ARN total por el método de Trizol™ (Life Technology, USA), junto con un choque térmico, siguiendo las indicaciones del fabricante. El ARN se trató con 1 U/μl de DNase 1 (ThermoFisher, USA).

Con el fin de verificar la ausencia de ADN genómico, las muestras de ARN se emplearon en una PCR convencional, utilizando cebadores específicos para el gen de la actina como gen normalizador (cuadro 2), en la cual la ausencia de un producto de amplificación garantiza la ausencia de ADN genómico. El ARN purificado fue cuantificado y analizado utilizando un Nanodrop 2000™ (ThermoFisher, USA).

**Cuadro 2:** Cebadores usados en el análisis de la PCR en tiempo real

Gen	Cebador	Secuencia	Tamaño del fragmento de ADN
ERG11*	*Forward* *Reverse*	5' CCATGTCCGAGCTCATCATTCTT 3' 5' ACTGGGAAGGGGCAAGTTGG 3'	150 pb
*AFR1+*	*Forward* *Reverse*	5' CCCACTTTGCCATACTTTTGG 3' 5'AACTGTGGAGACAAGACCACTGATAA 3'	85 pb
MDR1*	*Forward* *Reverse*	5'ACCCACTCTTTTCGGTAC 3' 5'TACCGCGCTCACCAAC 3'	180 pb
Actina*	*Forward* *Reverse*	5'CCAAGCAGAACCGAGAGAAGATG 3' 5'GGACAGTGTGGGTGACACCGT 3'	156 pb

* (17)

+ (18)

*Síntesis del ADN complementario (ADNc)*

La cadena complementaria de ADN (ADNc) se sintetizó a partir de los ARN totales extraídos, empleando una concentración 600 ng totales (3 pl de una solución a 200 ng/pl) de ARN, en una reacción de 20 μl de volumen final, usando el juego de reactivos comercial: Maxima H Minus First Strand cDNA Synthesis Kit™ (ThermoFisher, USA), según las instrucciones del fabricante.

*Reacción en cadena de la polimerasa en tiempo real*

La PCR en tiempo real se practicó usando el juego de reactivos comerciales Maxima SYBR Green/Fluorescein qPCR Master Mix™ (2X), siguiendo las indicaciones del fabricante (ThermoFisher, USA).

La reacción de PCR contenía 2 μl de ADNc, el cual se hallaba a una concentración de 300 ng/μl, 10 μl de SYBR Green Master Mix 2X, y 0,1 mM de cada cebador (cuadro 1). Las reacciones tuvieron un volumen final de 20 μl, el cual fue ajustado con agua libre de nucleasas. El equipo empleado para detectar la fluorescencia fue el CFX96 Real-Time PCR Detection System™ (Bio-Rad, Headquarters Hercules, California, USA).

Se hizo una desnaturalización inicial a 94 °C durante 5 minutos, seguida de 45 ciclos de PCR, de la siguiente manera: 95 °C durante 50 segundos, 60 °C por 20 segundos, y 72 °C durante 30 segundos. El análisis de las curvas de disociación (*melting curve analysis*) de los productos de PCR, se basó en la aplicación de un gradiente de temperaturas creciente con posterioridad a la PCR, para determinar la temperatura de fusión (Tm), que es la temperatura a la que el 50 % de las moléculas de ADN de doble cadena son desnaturalizadas.

Este paso se hizo después de la polimerización para descartar la posibilidad de amplificación no específica o de formación de dímeros de iniciadores. Los niveles de expresión del ARN mensajero (ARNm) se calcularon mediante un método de expresión relativa, usando la fórmula 2^MCT^, donde AACT es la diferencia entre el gen diana y el gen de la actina ^22^. Cada experimento se llevó a cabo en dos réplicas biológicas y cada muestra fue evaluada por triplicado.

## Resultados

Las pruebas de sensibilidad a los antifúngicos permitieron caracterizar los aislamientos según su fenotipo de sensibilidad al fluconazol. Como resultado de esta prueba, 8 aislamientos mostraron una sensibilidad disminuida con CIM mayores que los puntos de corte epidemiológicos (8 μg/ml); de estos 8 aislamientos, 5 obtuvieron valores de CIM mayores de 64 μg/ml y 3 presentaron valores de CIM entre 16 y 61 μg/ml. Además, 6 aislamientos mostraron valores de CMI menores que los puntos de corte epidemiológico (CIM entre 1 y 6 μg/ml) (cuadro 1).

### Perfiles de expresión de los genes ERG11, AFR1 y MDR1

Los controles empleados para verificar la ausencia de ADN en las muestras de ARNm demostraron la ausencia de productos de amplificación, verificándose la pureza del ARN extraído.

**Figura 1:**
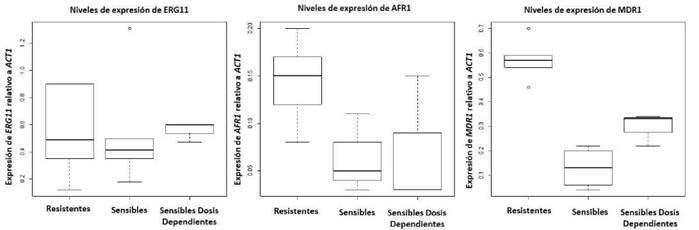
Efecto del fluconazol en la expresión de los genes *ERG11*, *AFR1* y *MDR1*. Los resultados de la expresión relativa de estos genes se calcularon usando la fórmula 2“^CT^ relativa al gen de la actina (ACT1), como gen normalizador ^19^. Las cajas representan el rango intercuartil o la media del 50 % de las observaciones.

Los niveles de ARNm de los genes *ERG11*, *AFR1 y MDR1* relativos a la actina (gen constitutivo), se evaluaron en todos los aislamientos registrados en el cuadro 1. La proporción de los niveles de ARNm del gen *ERG11* comparados con los del gen constitutivo *(ERG11* /*ACT1*), no fueron significativamente diferentes entre los aislamientos de los dos fenotipos de resistencia evaluados: sensibles, y con sensibilidad disminuida no obstante, en los aislamientos resistentes, la expresión relativa a la actina del gen *AFR1* fue 2,4 veces mayor que en los aislamientos sensibles, y la del gen *MDR1* fue 7,7 veces mayor que en el grupo con valores CIM entre 16 y 61 μg/ml (figura 1). Asimismo, el análisis de las curvas de fusión (*melting curves*), permitió descartar la presencia de productos de amplificación inespecíficos o dímeros entre las parejas de cebadores evaluadas.

## Discusión

El objetivo principal de este estudio fue determinar los perfiles de expresión de los genes *ERG11*, *AFR1 y MDR1.* La sobreexpresión del gen *ERG11*, que codifica para la enzima diana de los azoles (lanosterol 14 α-desmetilasa), constituye uno de los principales mecanismos por medio del cual las levaduras de importancia médica, incluidas las especies de *Cryptococcus*, adquieren resistencia a estos antifúngicos ^23^. Asimismo, la sobreexpresión de los genes *AFR1 y MDR1*, que codifican para las bombas de eflujo son factores importantes que le confieren resistencia a *C. neoformans* contra el fluconazol ^24^.

Los valores de la CIM muestran que existe una gran frecuencia de aislamientos colombianos con sensibilidad disminuida al fluconazol en población con sida (mayor de 50 %), similar a lo reportado por Agudelo, *et al*. (2015), quienes encontraron una tendencia similar utilizando un tamaño de muestra más grande (n=72). Lo anterior demuestra que los aislamientos recuperados de pacientes con sida son menos sensibles al tratamiento con fluconazol ^25^^,^^26^, y que se debe profundizar en el estudio de las causas de esta alta frecuencia en la subpoblación colombiana.

A pesar de no existir un consenso acerca de los puntos de corte epidemiológicos, los aislamientos de *C. neoformans var. grubii* considerados menos sensibles al fluconazol en este estudio, se categorizarían igual utilizando los puntos de corte reportados en la mayoría de los estudios publicados ^27^. Por tanto, la expresión de los genes *AFR1 y MDR1* podría compararse con la encontrada en otras poblaciones consideradas resistentes.

En algunos estudios se ha demostrado que ni el aumento en la magnitud de la expresión de los genes, ni las variaciones en la secuencia codificante del gen *ERG11,* pueden explicar los altos valores de CIM encontrados en aislamientos clínicos de *C. gattii*^23^. Resulta de suma importancia explorar este comportamiento, específicamente en *C. neoformans* var*. grubii*, y correlacionarlo con los hallazgos del presente estudio, pues, en todos los aislamientos evaluados bajo nuestras condiciones experimentales, la magnitud de la expresión registrada para el gen *ERG11* no se correlaciona con los perfiles de resistencias encontrados. Esto indica que es posible que las mutaciones en el *ERG11*, la sobreexpresión de otros genes *ERG* o el aumento de la actividad de las bombas de eflujo, sean responsables de la resistencia en estos aislamientos.

Así, las sustituciones de aminoácidos en los genes *ERG* pueden contribuir a producir una baja afinidad entre la enzima lanosterol 14-a-desmetilasa (Erg11p) y el fluconazol, causando la resistencia ^28^.

Por su parte, Florio, *et al.*^29^, reportaron un efecto compensatorio de aumento, entre 2,09 y 3,95 veces, de la expresión de ocho genes *ERG* (*ERG1*, *ERG2*, *ERG3*, *ERG5*, *ERG7*, *ERG11*, *ERG13 y ERG25*), bajo inducción con fluconazol durante cortos periodos ^29^. Esto concuerda con lo hallado en el presente estudio, en el cual, los valores de la expresión de

*ERG11* fueron similares en los tres grupos de sensibilidad aquí probados. Por tanto, se requieren nuevos estudios en que se evalúen otros genes de tipo *ERG*, con el fin de confirmar este comportamiento en los aislamientos colombianos de *C. neoformans* var. *grubii* obtenidos de pacientes con HIV.

A la fecha, *AFR1 y MDR1* son los únicos genes conocidos que codifican para dos bombas de eflujo asociadas con la resistencia de *C. neoformans* a medicamentos antifúngicos ^29^^,^^30^. Los estudios de bloqueo (*knock out*) de genes revelan que la disrupción del gen *AFR1* resulta en la deleción total del gen, lo cual confiere al mutante receptor total sensibilidad a los azoles y, además, la reintroducción del gen restaura el fenotipo de resistencia.

Estos hallazgos indican claramente que la sobreexpresión de *AFR1* es un factor determinante importante en el aumento de la resistencia *in vitro* de *C. neoformans* a los azoles. Sin embargo, es posible que otros mecanismos estén involucrados en este fenómeno ^18^.

En este mismo sentido, Sionov, *et al*., describen cierto mecanismo de adaptación del hongo como respuesta al antifúngico, denominado heterorresistencia, en el cual se observa que el número de cromosomas disómicos se correlaciona de manera positiva con la duración de la exposición al fluconazol; la duplicación del cromosoma 1 estuvo estrechamente relacionada con dos genes, *ERG11* y *AFR1*. Tal plasticidad genómica hace posible que las células sobrelleven el estrés causado por el efecto del medicamento, lo cual se ha observado en el serotipo A (*C. neoformans* var*. grubii*) y en el D (*C. neoformans*) ^31^.

En resumen, hemos demostrado que los genes *AFR1 y MDR1* de *C. neoformans* var*. grubii* presentan una sobreexpresión en aislamientos resistentes al fluconazol, mientras que el gen *ERG11* mantiene homogénea su expresión en todos los aislamientos de *C. neoformans* var*. grubii,* de los tres fenotipos de resistencia evaluados. Lo anterior sugiere que estas bombas de eflujo contribuyen a la resistencia al fluconazol en este grupo de aislamientos estudiados.

No obstante, los patrones de resistencia que se registran en este hongo, sumados a los casos de recaídas en pacientes con HIV, no pueden atribuirse únicamente a casos de resistencia por exposición al fármaco, debido a que la heterorresistencia es un mecanismo alternativo con un gran potencial en este proceso. En el presente estudio, se incluyó únicamente una variante de cada gen evaluado y una muestra de 14 aislamientos. El análisis de los niveles de expresión de otros genes de tipo *ERG*, así como la variante *AFR2 y* la inclusión de un mayor número de aislamientos con el fenotipo de heterorresistencia, permitirán elucidar de manera más clara la función de estos genes en el complejo mecanismo de resistencia a los azoles en *Cryptococcus* patógenos.
